# Metagenomic screening of the virome of symptomatic tomato plants from La Réunion Island uncovers a complex of viruses including a newly identified whitefly-transmitted polerovirus

**DOI:** 10.1007/s00705-025-06426-y

**Published:** 2026-01-25

**Authors:** Jean-Michel Lett, Sarah Scussel, Sélim Ben Chéhida, Murielle Hoareau, Denis Filloux, Emmanuel Fernandez, Philippe Roumagnac, Evelyne Parvedy, Elise Quirin, Clarisse Clain, Janice Minatchy, Estelle Roux, Pierre-Yves Teycheney, Pierre Lefeuvre

**Affiliations:** 1 CIRAD, UMR PVBMT, Pôle de Protection des Plantes, F-97410 Saint-Pierre, Ile de La Réunion France; 2 CIRAD, UMR PVBMT, Direction de l’Agriculture, AGROPOL, cellule Recherche-Innovation-Valorisation, Papara, Tahiti Polynésie Française; 3 Université de La Réunion, UMR PVBMT, Pôle de Protection des Plantes, F-97410 Saint-Pierre, Ile de La Réunion France; 4 CIRAD, UMR PHIM, 34398 Montpellier, France; 5 PHIM Plant Health Institute, Univ Montpellier, CIRAD, INRAE, Institut Agro, IRD, Montpellier, France; 6 FDGDON, Pôle de Protection des Plantes, F-97410 Saint-Pierre, La Réunion France; 7 CIRAD, UMR PVBMT, College of Agriculture, Can Tho University, Can Tho, Vietnam

## Abstract

Using unbiased high-throughput sequencing for metagenomic screening of viruses in diseased tomato plants, we identified a viral complex that includes viruses previously reported in tomato crops on La Réunion Island as well as a novel polerovirus, tentatively named "tomato necrotic yellowing virus" (ToNYV, proposed species, "*Polerovirus ToNYV*"). Molecular characterization and phylogenetic analysis revealed that ToNYV is closely related to two recently described poleroviruses from Africa and the Middle East, one of which is transmitted by the whitefly *Bemisia tabaci*, a trait uncommon among poleroviruses. Our transmission experiments demonstrated that ToNYV is also transmitted by *B. tabaci* and is prevalent across major tomato-growing regions of La Réunion. These findings highlight the value of metagenomic virome analysis in diseased plants for identifying novel viruses potentially involved in emerging plant diseases, either individually or as components of viral complexes.

**Supplementary Information:**

The online version contains supplementary material available at 10.1007/s00705-025-06426-y.

Recent advances in high-throughput sequencing (HTS) technologies and bioinformatics have transformed the field of plant virology, particularly in the detection and diagnosis of viral pathogens of plants [1]. Viral metagenomics, referred to as viromics, enables comprehensive profiling of viral communities in plant samples by detecting both known and novel viral sequences. Viromics has matured significantly, with various enrichment strategies now available to enhance the recovery of viral nucleic acids. These include total RNA sequencing with ribosomal RNA (rRNA) depletion [2], extraction of virion-associated nucleic acids (VANAs) from viral particles [3], isolation of double-stranded RNA (dsRNA) [4], and sequencing of virus-derived small interfering RNAs (siRNAs) [5]. As this methodology has gained widespread acceptance by the scientific community, it has proven highly effective in identifying plant viruses across both wild and cultivated ecosystems [4–7]. Viromics is now increasingly regarded as the method of choice in complex epidemiological contexts, particularly when the etiological agents are unknown or poorly characterized.

One such epidemiological scenario involved viral diseases affecting tomato crops on La Réunion Island. Since the 1990s, these crops have faced a series of successive biological invasions involving various viruses and their insect vectors. Notable examples include tomato spotted wilt virus (TSWV, species *Orthotospovirus tomatomaculae*, genus *Orthotospovirus*, family *Tospoviridae*) and its thrips vector *Frankliniella occidentalis* [8]; potato virus Y (PVY, species *Potyvirus yituberosi*, genus *Potyvirus*, family *Potyviridae*) and its aphid vectors *Myzus persicae*, *Aphis gossypii,* and *A. fabae* (PRPV database, https://db.e-prpv.org/) [9]; and both the Mild (TYLCV-Mld [10]) and Israel (TYLCV-IL [11, 12]) strains of tomato yellow leaf curl virus (TYLCV, species *Begomovirus coheni*, genus *Begomovirus*, family *Geminiviridae*) and their whitefly vector *Bemisia tabaci* MEAM1 (formerly known as biotype B) [13]. Additionally, tomato chlorosis virus (ToCV, species *Crinivirus tomatichlorosis*, genus *Crinivirus*, family *Closteroviridae*) [13], transmitted by both *B. tabaci* and *Trialeurodes vaporariorum* [13], has been reported, as well as southern tomato virus (STV, species *Amalgavirus lycopersici*, genus *Amalgavirus*, family *Amalgaviridae*) [14] for which no evidence of horizontal transmission has been reported [15].

In 2015, two tomato leaf samples displaying a mixture of symptoms characteristic of TYLCV and ToCV infections were collected on Reunion Island, one from a field-grown plant (C15-7) and one from a greenhouse-grown plant (C15-10). The viral diversity in these samples was investigated using a VANA-based metagenomic approach [3]. Libraries were prepared from purified amplicons and sequenced by Genewiz (USA) on an Illumina HiSeq device using a 2x250 bp configuration. Reads were demultiplexed using cutadapt 1.18 [16] with a minimum overlap of 10 nt and other parameters set to default. After quality control of demultiplexed reads using Trimmomatic v0.35 [17] (parameters, SLIDINGWINDOW:5:20 and MINLEN:80), a total of 59,182 and 92,526 quality-controlled reads were obtained for sample C15-7 and sample C15-11, respectively (Supplementary Table S1). Reads were then assembled *de novo* into contigs using SPAdes v3.13.0 [18]. BLASTn and BLASTx comparisons of the resulting contigs against viral sequences in the GenBank database revealed that nine contigs (>500 nt in length) from sample C15-7 and 16 from sample C15-11 were potentially derived from plant RNA or DNA viruses. These contigs showed similarity to genome sequences from viruses from several families, including *Amalgaviridae*, *Closteroviridae*, *Geminiviridae*, *Potyviridae*, and the former family *Luteoviridae* (Supplementary Table S1).

In plant C15-7, two contigs (Contig-1395, 1,935 nt, and Contig-1396, 1,418 nt) shared the highest nucleotide sequence similarity with TYLCV-IL, showing 99.1% and 98% identity to sequences AM409201 and FJ012359, respectively (Supplementary Table S2). Additionally, three contigs (Contig-1401, 1332, and 900, ranging from 1,077 to 3,066 nt) showed 90.5% to 91.5% nucleotide sequence identity to PVY (family *Potyviridae*, genus *Potyvirus*) sequences (AM409201 and FJ012359).

From plant C15-11, five contigs (Contig-5040, 5146, 3288, 5051, and 3286, ranging in size from 1,253 to 2,485 nt) displayed 98% to 99% nucleotide sequence identity with RNA1 of ToCV (family *Closteroviridae*, genus *Closterovirus*) sequences (KJ740256 and KY471129). Another five contigs (Contig-5139, 5035, 3282, 5031, and 5145, ranging in size from 2,224 to 3,788 nt) shared 96.7% to 97.5% identity with ToCV RNA2 sequences (KP137101, KY810787, and KY471130). Two additional contigs (Contig-5036, 968 nt, and Contig-3280, 536 nt) showed 98% and 99.8% identity, respectively, to a southern tomato virus (STV, family *Amalgaviridae*, genus *Amalgavirus*) sequence (MN216389).

The nucleotide and amino acid sequence identity of these contigs to reference sequences in the GenBank database exceed the species demarcation thresholds recommended for their respective genera: 65–70% amino acid sequence identity in the RNA-dependent RNA polymerase (RdRp) for the genus *Amalgavirus* [19], https://ictv.global/report/chapter/amalgaviridae/amalgaviridae; 75% amino acid sequence identity in the RdRp protein, heat shock protein 70 homologue (HSP70h), and coat protein (CP) for the genus *Closterovirus* [20]; 91% nucleotide sequence identity in the complete DNA-A component for the genus *Begomovirus* [21]; and 80% amino acid sequence identity in the CP for the genus *Potyvirus* [22]. These results indicate that the viral isolates from which these partial genomic sequences were derived are members of the established virus species *Amalgavirus lycopersici* (STV), *Crinivirus tomatichlorosis* (ToCV), *Begomovirus coheni* (TYLCV), and *Potyvirus yituberosi* (PVY).

In contrast, analysis of plant C15-7 revealed four contigs (Contig-894, 1392, 1390, and 897) ranging in size from 1,755 to 4,218 nt, that shared the highest nucleotide sequence identity (82.3% to 86.6%) with sequences of African eggplant yellowing virus (AeYV; accession no. KX856972), a member of the genus *Polerovirus* (family *Solemoviridae*) (Supplementary Table S2). Similarly, from plant C15-11, four contigs (Contig-5034, 5142, 5140 and 3283) ranging in size from 1,990 to 4,703 nt, exhibited 75.5 to 80.8% nucleotide sequence identity to another AeYV sequence (accession no. KX856971). Within each sample, the AeYV-related contigs exhibited large overlaps with one another, with minimum pairwise identity values of 97.5% and 99.1% for contigs obtained from plant C15-7 and plant C15-11, respectively.

To obtain the complete genome sequence of the AeYV-related virus, a tomato sample (C20-19) showing symptoms of necrosis, yellowing, and reddening of the leaves (Fig. 1) that tested positive for the AeYV-related virus and negative for ToCV and PVY by RT-PCR, and negative for TYLCV by PCR, was subjected to Illumina RNA sequencing (2x150-bp paired-end reads on an Illumina HiSeq platform) following ribosomal RNA depletion (GeneWiz, Leipzig, Germany). Quality control of the raw reads using Trimmomatic v0.35 [17] (parameters, SLIDINGWINDOW:5:20 and MINLEN:80) resulted in ~11.4 million paired-end reads. Among the contigs obtained after *de novo* assembly using MEGAHIT v1.2.9 [23], a single viral contig of 5,953 nt with an average coverage of 5004-fold was identified through searches against the NCBI gbvrl viral database using DIAMOND 0.9.22 with an e-value cutoff of <10^−5^ [24]. No other viral sequences were detected in sample C20-19.

**Fig. 1:**
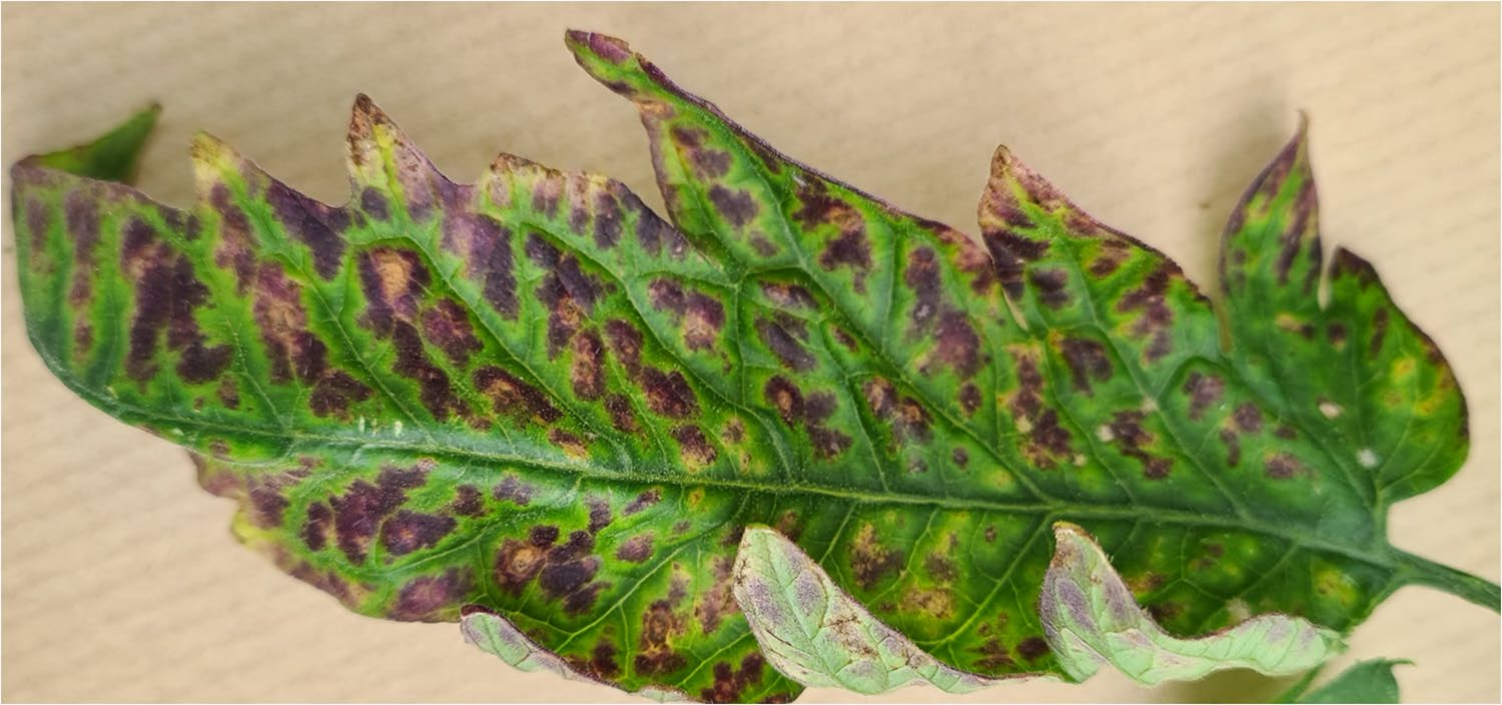
Symptoms of tomato necrotic yellowing disease. Necrotic yellowing and reddening symptoms were observed on the leaves of a greenhouse-grown tomato plant associated with the presence of tomato necrotic yellowing virus (ToNYV). The sample used for complete genome sequencing of isolate RE-C20-19–20 was collected from this plant. For detailed sample information, refer to Supplementary Table S4

The low-coverage region corresponding to the readthrough domain between ORF3 and ORF5 was confirmed by Sanger sequencing of RT-PCR amplicons obtained using primers AeYV-Like-F3988 and AeYV-Like-R4795 (Supplementary Table S3). The sequence of the 5’-terminal region was verified using both (1) the MinION sequencing strategy with the strand-switching method described by Filloux et al. [25] and (2) the rapid amplification of cDNA ends strategy with viral sequence-specific reverse primers (Supplementary Table S3) followed by direct Sanger sequencing in both directions (Macrogen Europe, The Netherlands), as described in Orfanidou et al. [26]. Likewise, the sequence of the 3’-terminal region was verified using MinION sequencing after poly(A)-tailing, following the method of Wongsurawat et al. [27].

Integration of Illumina and MinION data resulted in a final consensus sequence of 5,995 nt for the RE-C20-19–20 isolate of the AeYV-related virus, which was deposited in the GenBank database under the accession number PV289033. BLASTn analysis revealed that the AeYV-related virus genome sequence shared the highest nucleotide sequence similarity (86.9% identity with 88% query coverage) with the eMA4 isolate of AeYV (KX856972). The AeYV-related virus genome exhibits the typical organization of poleroviruses, containing six open reading frames (ORFs), as predicted using the ORFfinder tool available on the NCBI website (Fig. 2A). The 5’ and 3’ termini of the genome begin with the nucleotides ACAAA and end with GT, respectively. A predicted −1 ribosomal frameshift for the P1-P2 fusion protein was identified at position 1,682, involving a conserved “slippery heptamer” sequence (GGGAAA**C**GGGAAA). The conserved sequence flanking the leaky stop codon separating the coat protein (P3, ORF3) from the readthrough domain (RTD) (P5, ORF5) (CCCAAA**TAG**GTAGA, with the stop codon in bold) was also present.

**Fig. 2:**
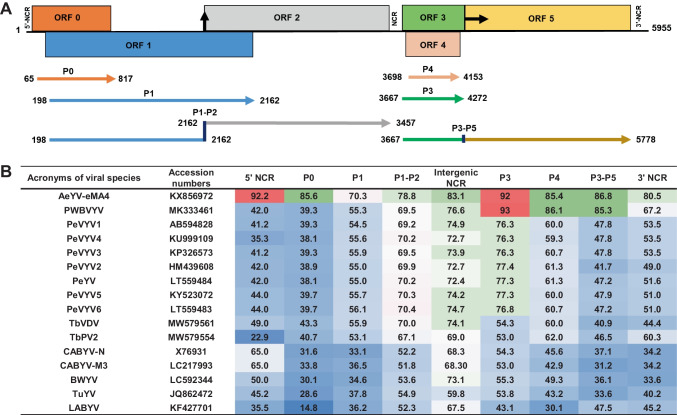
(**A**) Genome organization of tomato necrotic yellowing virus (ToNYV-[RE-C20-19–20]). Putative ORFs are shown as coloured boxes, with corresponding protein products represented by coloured arrows. The 5’ and 3’ ends of the genome are indicated. A vertical black arrow marks the −1 ribosomal frameshift site for the P1-P2 fusion protein at nucleotide position 1682, while a horizontal black arrow indicates the leaky stop codon at the end of ORF3. (**B**) Amino acid (aa) sequence identity of the encoded proteins and nucleotide sequence identity of the non-coding regions (NCRs) of ToNYV-[RE-C20-19–20] and closely related poleroviruses. Sequence similarity is indicated by color saturation: red for high similarity (90–100% identity), white/green for moderate similarity (70–90% identity), and blue for low similarity (<70% identity). Virus abbreviations are listed in Supplementary Table S4

To clarify the phylogenetic relationship between the AeYV-related virus and other poleroviruses, a maximum-likelihood (ML) phylogenetic tree was constructed using MEGA X software [28], based on a multiple sequence alignment performed using MUSCLE and the automatic selection of the best-fit nucleotide substitution model (GTR+G). Pairwise nucleotide sequence identity comparisons were performed using SDT v1.2 with pairwise deletion of gaps [29]. The resulting ML phylogenetic tree and the pairwise nucleotide identity matrix (Fig. 3) revealed that the complete genome sequence of the RE-C20-19–20 isolate clustered closely with the eMA4 isolate of AeYV from Mali (83% nucleotide sequence identity). Together, AeYV-related virus-RE-C20-19–20 and AeYV-eMA4 isolates form a distinct cluster positioned outside the pepper vein yellows virus (PeVYV) clade.

**Fig. 3:**
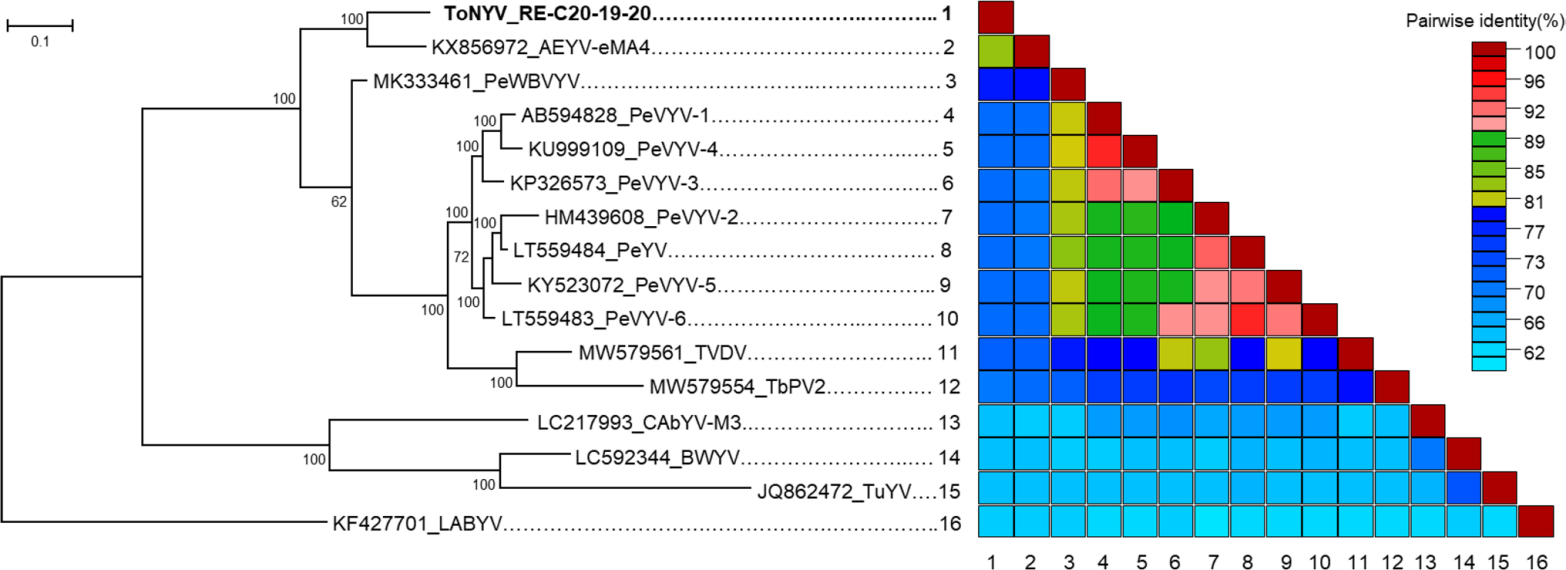
Maximum-likelihood phylogenetic analysis and pairwise nucleotide identity matrix. A maximum-likelihood phylogenetic tree was constructed based on genome sequence alignments of tomato necrotic yellowing virus (ToNYV) isolate RE-C20-19–20 and selected poleroviruses. Bootstrap values above 70% (based on 1,000 replicates) are displayed at the corresponding nodes. Adjacent to the tree, a colour-coded matrix illustrates the percentage identity between two viruses. The colour key on the right indicates the correspondence between colours and identity ranges. For virus abbreviations, refer to Supplementary Table S4

Amino acid (aa) sequence comparisons of proteins encoded in the genome of isolate RE-C20-19–20 with those of closely related poleroviruses (Fig. 2B) were performed using MEGA X [28]. Proteins encoded in the 3’ region of the genome (P3, coat protein,P4, movement protein; and the P3-P5 readthrough fusion protein) shared the highest sequence similarity (85.3% to 93% identity) with their counterparts in AeYV and pepper whitefly-borne vein yellows virus (PeWBVYV). In contrast, proteins encoded in the 5’ half of the genome (P0, RNA silencing suppressor; P1, serine protease; and P1-P2 fusion, RNA-dependant RNA polymerase) displayed lower sequence identity values, ranging from 70.3% to 85.6%, with the highest similarity observed with AeYV. The highest aa sequence similarity was found in the P3 protein, showing 93% and 92% identity to AeYV (KX856971) and PeWBVYV (MK333461), respectively (Fig. 2B). The lowest similarity (70.3% sequence identity) was observed for the P1 protein when compared to its counterpart in AeYV (KX856971). Except for the P3 protein, all of the AeYV-related viral proteins exhibited more than 10% aa sequence divergence relative to known poleroviruses, thus exceeding the species demarcation threshold defined for the genus *Polerovirus* [30]. These findings support the identification of a putative novel polerovirus, which we have tentatively named "tomato necrotic yellowing virus" (ToNYV).

Recombination analysis using RDP4 software [31], with default parameters and a full-genome sequence alignment of ToNYV and other poleroviruses did not reveal any evidence of recombination in the ToNYV genome.

Poleroviruses were traditionally thought to be transmitted exclusively by aphids in a persistent and circulative manner [32]. However, recent discoveries have identified two poleroviruses, PeWBVYV [33] and the Brazilian recombinant isolates of cucurbit aphid-borne yellows virus (CABYV) [34], that are transmitted by whiteflies. Given the near absence of aphids and the widespread presence of whiteflies in tomato greenhouses affected by ToNYV, we investigated the vector transmission capacity of ToNYV by the aphid *Myzus persicae* and the whitefly *Bemisia tabaci* MEAM1. For transmission assays, approximately 400 aphids and 1,200 whiteflies, reared on tobacco and cabbage, respectively, were given a 48-hour acquisition period on ToNYV-infected tomato leaves exhibiting strong ToNYVD symptoms. Leaves were collected from a 5-month-old greenhouse tomato plant showing severe symptoms of ToNYVD. The presence of ToNYV genomic RNA was confirmed by RT-PCR, and the absence of ToCV and PVY genomic RNA and TYLCV genomic DNA was confirmed by specific RT-PCR and PCR, respectively. Subsequently, these aphids and whiteflies were transferred to separate rearing cages and allowed a 48-hour inoculation access period on 10 and 20 15-day-old tomato seedlings (cv. Roma), respectively. No symptoms of ToNYVD were observed, and no ToNYV genomic RNA was detected by RT-PCR in any of the 10 tomato plants 30 days after they were exposed to viruliferous *M. persicae*, indicating that this aphid species may not transmit ToNYV. In contrast, 35% (7/20) of the tomato plants exposed to viruliferous *B. tabaci* developed yellowing symptoms 30 days post-exposure, five of which tested positive for ToNYV by RT-PCR followed by Sanger sequencing. These findings confirm that ToNYV is transmissible by *B. tabaci* MEAM1.

To further investigate the distribution of ToNYV, a virus survey was conducted in 2018 and 2020 in tomato greenhouses located in the main tomato-producing regions of southern and south-eastern La Réunion (Supplementary Table S5). Leaf samples exhibiting virus-like symptoms, including leaf yellowing, reddening, necrosis, deformation, and stunting, were collected from multiple commercial cultivars. The presence of ToNYV genomic RNA was assessed using RT-PCR on total RNA extracted from symptomatic leaf tissues using an RNeasy Plant Mini Kit (QIAGEN, France), following the manufacturer’s protocol. Amplicons were sequenced directly in both directions (Macrogen Europe, The Netherlands). RT-PCR was performed using the degenerate primers Polero-FD3249 and Polero-RD4339 (Supplementary Table S3), which were designed in this study to cover the ORF2 and ORF3 regions, encoding the polerovirus RNA-dependent RNA polymerase (RdRp) and the coat protein (CP), respectively. These regions were identified in contigs obtained through the VANA-based metagenomics approach, as well as sequences from emerging poleroviruses such as AeYV [35], PeVYV-1 to −6 [21, 36], and PeWBVYV [33]. Reverse transcription was carried out using a RevertAid RT Reverse Transcription Kit (Thermo Fisher Scientific, France) with 1 µM downstream primer and 1 µM random hexamers, following the manufacturer’s instructions. Briefly, 5 μl of extracted RNA was added to a reaction mixture consisting of 1 μl of random hexamer primers and 6 μl of diethylpyrocarbonate (DEPC)-treated water. This mixture was incubated at 70°C for 5 min for RNA denaturation and then placed on ice for an additional 5 min. A second reaction mix containing 4 μl of reaction buffer, 1 μl of Ribolock RNase Inhibitor, 2 μl of 10 mM dNTP mix, and 1 μl of reverse transcriptase (RevertAid H Minus M-MuLV Reverse Transcriptase) at a concentration of 200 U/μl was prepared and added to the first reaction mix. Negative controls were included in each set of RT reactions in order to detect potential contamination. PCR amplification was performed using a GoTaq G2 DNA Polymerase kit (Promega, France), following the manufacturer’s instructions. Reactions were prepared in a final volume of 25 µL: 5 µL of GoTaq Flexi Buffer, 2.5 µL of 10 mM dNTP mix, 1.5 µL of 25 mM MgCl_2_, 1 µL of forward primer, 1 µL of reverse primer, 0.2 µL of GoTaq G2 Flexi DNA Polymerase (5 U/µL), 11.8 µL ultrapure water, and 2 µL of DNA sample. The cycling conditions used were 94 °C for 5 min, followed by 35 cycles of 94 °C for 1 min, 55 °C for 1 min, 72 °C for 30 s and a final extension at 72 °C for 5 min. The amplified PCR products were analysed on a 1% agarose gel to confirm the presence of amplicons of the expected size and sequenced in both directions by the Sanger method at Macrogen Europe (The Netherlands).

ToNYV genomic RNA was detected in 21% (8/38) of the samples collected in 2018 and 14.6% (14/96) of those collected in 2020. Positive samples were distributed across four localities (Saint-Louis, Saint-Pierre, Saint-Joseph, and Saint-Philippe; Supplementary Table S5), covering the southern and south-eastern parts of the island’s main vegetable production zone. These finding indicate a widespread presence of ToNYV within the tomato-growing production areas of La Réunion.

In conclusion, we demonstrate the utility of high-throughput-sequencing-based metagenomic screening for the detection and characterisation of plant viruses in symptomatic tomato plants from fields and greenhouses in La Réunion. Alongside previously reported tomato-infecting viruses in La Réunion (TSWV, PVY, TYLCV, ToCV, and STV), we identified and characterised a novel polerovirus associated with necrotic reddening and yellowing of leaves. The complete genome sequence of this virus was obtained and analysed. Based on its distinct genomic organisation, amino acid sequence divergence, phylogenetic placement, and confirmed transmission by *B. tabaci*, we propose that this virus represents a new species within the genus *Polerovirus*, for which we suggest the species name "*Polerovirus ToNYV*".

As noted by Ghosh et al. [33], the emergence of whitefly-transmitted poleroviruses poses a significant threat to global agriculture, particularly because *B. tabaci* is regarded as a “supervector” of plant viruses. Some of its cryptic species, such as MEAM1 and MED, exhibit extreme polyphagy and high levels of insecticide resistance [37]. The specificity of polerovirus transmission by aphid vectors is largely determined by the N-terminal region of the readthrough domain (RTD) [32, 38]. While the vector of AeYV remains unconfirmed, the consistent association of AeYV-infected tomato crops in Côte d'Ivoire with whitefly populations [39], together with the high degree of sequence similarity in the CP and RTD regions between AeYV, ToNYV, and PeWBVYV, strongly suggests that AeYV may also be transmitted by *B. tabaci*. The recent emergence of these novel poleroviruses across geographically distant regions, including the Middle East, South America, West Africa, and the Mascarene Archipelago, raises important questions regarding their evolutionary trajectories and possible common origins. However, the apparent lack of homology in factors that are likely to determine transmission specificity (CP and RTD) between African poleroviruses (AeYV, ToNYV and PeWBVYV) and the South American recombinant isolates of CABYV, which are also transmitted by *B. tabaci*, suggests distinct evolutionary lineages and a convergent acquisition of this transmission ability. Clarifying these patterns will require in-depth studies on the ecology, vector interactions, and phylogeography of these viruses, as such knowledge is essential to identify their dissemination routes and to evaluate their potential threat to global tomato production.

## Supplementary Information

Below is the link to the electronic supplementary material.

Supplementary file1 (XLSX 16 KB)

Supplementary file2 (XLSX 16 KB)

Supplementary file3 (XLSX 10 KB)

Supplementary file4 (XLSX 14 KB)

Supplementary file5 (XLSX 18 KB)

## Declarations

### GenBank accession number

The complete genome sequence reported in this manuscript has been deposited in the GenBank database under the accession number PV289033 (ToNYV isolate RE-C20-19-20).
